# Epigenetic reprogramming drives endothelial dysfunction via neuropilin-1 in pulmonary hypertension

**DOI:** 10.1186/s10020-025-01386-0

**Published:** 2025-12-16

**Authors:** Maria T. Ochoa, Celine Leppert, Benedetta Ricchi, Elizabeth Singh, Marcello Rota, Malik Bisserier

**Affiliations:** 1 Department of Cell Biology and Anatomy, New York Medical College, Valhalla, NY 10595 USA; 2 Department of Physiology, New York Medical College, Valhalla, NY 10595 USA

**Keywords:** Histone acetylation, Pulmonary arterial hypertension, Endothelial dysfunction, Epigenetics, Gene expression

## Abstract

**Background:**

Pulmonary arterial hypertension (PAH) is characterized by progressive vascular remodeling and right ventricular failure. Endothelial dysfunction plays a key role in PAH initiation and progression. Recent studies have implicated epigenetic dysregulation, particularly histone acetylation, as a key contributor to the transcriptional reprogramming underlying PAH. Among the histone acetyltransferases, EP300 catalyzes H3K27 acetylation and modulates transcriptional and metabolic programs in vascular cells. However, its specific role in endothelial dysfunction in PAH remains unclear.

**Methods and results:**

We investigated the role of EP300 in regulating transcriptional and functional responses in human pulmonary artery endothelial cells (PAECs) in PAH. Human and experimental PAH tissues showed increased EP300 expression and elevated H3K27ac levels. Pharmacological inhibition of EP300 in PAH-derived PAECs reduced global H3K27ac levels, oxidative stress, abnormal proliferation, and inflammatory responses. Transcriptomic analysis revealed that EP300 regulates gene networks related to inflammation, angiogenesis, and metabolism. Among these, neuropilin-1 (NRP1) has emerged as a direct EP300 target marked by H3K27ac enrichment in its regulatory regions. EP300 overexpression up-regulated NRP1 expression, whereas EP300 inhibition decreased NRP1 expression. Functional studies further demonstrated that NRP1 modulates VEGFR2 signaling and glycolytic gene expression. Notably, the pharmacological inhibition of NRP1 with EG00229 attenuated EP300-induced endothelial proliferation and oxidative stress in vitro.

**Conclusions:**

Our findings identified EP300 as a central epigenetic regulator of endothelial dysfunction in PAH through H3K27ac-dependent activation of *NRP1*. The EP300-NRP1 axis integrates inflammatory, angiogenic, and metabolic signaling, contributing to endothelial dysfunction and disease onset. Targeting EP300-mediated histone acetylation may represent a promising therapeutic strategy for restoring endothelial homeostasis and slowing disease progression in patients with PAH.

**Supplementary Information:**

The online version contains supplementary material available at 10.1186/s10020-025-01386-0.

## Introduction

Pulmonary arterial hypertension (PAH) is a rare yet devastating vascular disease characterized by sustained elevation of pulmonary arterial pressure, increased pulmonary vascular resistance, and right ventricular failure, if left untreated (Boucherat et al. 2025; Weatherald et al. 2024; Kovacs et al. 2024). PAH remains incurable, with a 3-year survival rate of less than 60%, and lung transplantation is the only definitive intervention in advanced stages. Pathophysiologically, PAH is driven by extensive remodeling of the distal pulmonary arteries, characterized by endothelial dysfunction, medial hypertrophy, adventitial fibrosis, and the formation of occlusive lesions (Evans et al. 2021; Budhiraja et al. 2004). Endothelial dysfunction is a hallmark of PAH pathogenesis. Pulmonary artery endothelial cells (PAECs) transition from a quiescent to a proliferative, apoptosis-resistant, and pro-inflammatory phenotype (Kurakula et al. 2021; Correale et al. 2024; Ranchoux et al. 2018). However, the molecular mechanisms underlying endothelial dysfunction in PAH remain unclear.

Emerging evidence suggests that epigenetic regulation plays a central role in PAH (Huston and Ryan 2016; Bisserier et al. 2020; Mitra et al. 2024; Dave et al. 2023; Jankowski et al. 2023). Histone acetyltransferases have recently emerged as key modulators of transcriptional identity in PAH (Bisserier et al. 2020; Dave et al. 2023; Jankowski et al. 2023; Czerwinski et al. 2025). EP300 lysine acetyltransferase (EP300, also known as KAT3B) is a transcriptional activator responsible, in part, for acetylation of lysine 27 on histone H3 (H3K27) (Cohen et al. 2011). This epigenetic modification reduces nucleosome compaction by neutralizing the positive charges on histone tails, thereby increasing chromatin accessibility (Bannister and Kouzarides 2011). H3K27ac facilitates the recruitment of bromodomain-containing coactivators, mediator complexes, and RNA polymerase II, thereby enabling efficient transcriptional initiation (Beacon et al. 2021; Ladurner et al. 2003). It plays a critical role in spatiotemporal regulation of gene expression during development, lineage specification, and environmental adaptation (Sun et al. 2025; Rada-Iglesias et al. 2011; Chen et al. 2012; Creyghton et al. 2010; Zentner et al. 2011; Kim et al. 2022). Increasing evidence suggests that aberrant deposition of H3K27ac contributes to transcriptional reprogramming in diseases such as cancer and pulmonary vascular remodeling diseases by integrating inflammatory, hypoxic, and metabolic signals (Yang et al. 2023; Chelladurai et al. 2022). Previous studies have demonstrated that EP300 is up-regulated in vascular cells of PAH patients (Chelladurai et al. 2022). Pharmacological inhibition of EP300 in precision-cut lung slices reduces vascular remodeling (Chelladurai et al. 2022). Similarly, its inhibition ameliorated the hemodynamic and histological features of the disease in multiple preclinical models of PAH (Chelladurai et al. 2022). Notably, increased EP300 expression and global H3K27ac levels have been observed in remodeled pulmonary arteries, including endothelial-enriched compartments (Chelladurai et al. 2022). However, the extent to which EP300 orchestrates endothelial-specific transcriptional programs that initiate or perpetuate vascular dysfunction in PAH has not yet been investigated.

In this study, we investigated the role of histone acetyltransferase EP300 as a key epigenetic regulator of endothelial dysfunction in PAH. Specifically, we aimed to (1) define the endothelial-specific contribution of EP300 to PAH pathogenesis, (2) elucidate how EP300-mediated H3K27 acetylation drives transcriptional activation and endothelial dysfunction, and (3) evaluate the therapeutic potential of targeting EP300 in PAH-derived PAECs. Collectively, our findings identified EP300-driven epigenetic reprogramming as a central mechanism underlying pro-inflammatory, angiogenic, and metabolic alterations in the pulmonary endothelium. These findings establish that EP300 is a critical mediator of endothelial pathology and a potential therapeutic target.

## Material and methods

### Human lung tissues

Human lung specimens were obtained from the Pulmonary Hypertension Breakthrough Initiative (PHBI, Protocol# 02691), a multicenter biorepository dedicated to advancing translational research on PAH through the standardized acquisition of clinically annotated biospecimens. Lung tissues from patients with idiopathic PAH (IPAH) were harvested at the time of transplantation. Non-diseased donor lungs deemed unsuitable for transplantation were used as healthy controls. All tissue collection protocols were approved by the Institutional Review Boards (IRBs) at New York Medical College and strictly adhered to ethical guidelines. To ensure confidentiality, all the samples were fully deidentified.

### Experimental PAH models

Rodent models of PAH were established using the Sugen/hypoxia (SuHx) protocol in mice and the monocrotaline (MCT) model in rats, in accordance with institutional IACUC guidelines. Male C57BL/6 J mice (8–10 weeks old) received weekly subcutaneous injections of SU5416 (20 mg/kg) for 3 weeks, each followed by continuous exposure to normobaric hypoxia (10% O₂). After hypoxia, mice recovered for 7 days under normoxic conditions. In parallel, male Sprague–Dawley rats (200–225 g) were administered a single subcutaneous dose of monocrotaline (60 mg/kg) and monitored for 3–5 weeks. PAH development in both models was validated by right heart catheterization to measure right ventricular systolic pressure and mean pulmonary artery pressure (mPAP), as well as by calculation of the Fulton index (RV/[LV + S]) for right ventricular hypertrophy. Pulmonary vascular remodeling was confirmed histologically. Lung tissues were harvested for RNA extraction and gene expression analysis by QPCR.

### Cell culture

Human pulmonary artery endothelial cells (PAECs) and pulmonary artery smooth muscle cells (PASMCs) were obtained from PHBI (Protocol# 02691), a consortium dedicated to providing tissue specimens from patients with PAH and controls (failed donors, FD). Briefly, PAECs were cultured in complete endothelial cell medium (ScienCell, Cat# 1001) supplemented with endothelial cell growth supplement (ScienCell, Cat#1052), and maintained under standard conditions at 37 °C and 5% CO₂. Similarly, PASMCs were cultured according to the manufacturer’s protocol. Cells were maintained in Smooth Muscle Cell Medium (ScienCell, Cat# 1101), supplemented with 5% fetal bovine serum and smooth muscle cell growth supplements (ScienCell, Cat#1152). The cells were expanded to 80–90% confluence before being subcultured at a density of at least 5.0 × 10^5^ cells per 100 mm dish. Only the cells from passages 2 to 6 were used. Additional testing confirmed that all cell lines were free of mycoplasma and major viral or microbial contaminants, including bacteria, yeast, fungi, HIV-1, hepatitis B virus, and hepatitis C virus.

### Reagents and constructs

PAECs were treated with A-485 (MedChemExpress, Cat# HY-107455), a specific catalytic inhibitor targeting histone acetyltransferases EP300 and CBP. A-485 competitively binds to the acetyl-CoA site of p300/CBP. EG00229 trifluoroacetate (MedChemExpress, Cat# HY-10799, purity: 98.03%), is an antagonist of neuropilin 1 (NRP1) receptor. EG00229 specifically blocks the binding of VEGF-A to the b1 domain of NRP1.

### Transient overexpression

For transient overexpression experiments, human EP300 (Horizon Discovery, Cat# MHS1010–202,699,710) was introduced into FD-PAECs using Lipofectamine 2000 (Thermo Fisher, Cat# 11668019), according to the manufacturer’s guidelines. The experiments were conducted in 10 cm dishes containing 2 µg of plasmid DNA. An empty pCMV-SPORT-βgal vector (Thermo Fisher, Cat#10586014) served as the negative control. After 72 h of transfection, the cells were harvested for total RNA and protein extraction.

### siRNA-mediated gene silencing

PAH-PAECs were treated with Accell siRNA, specifically targeting EP300 (Horizon Discovery, Cat# A-003486–19-0005). Non-targeting siRNA (Horizon Discovery, Cat# D-001910–01–05) was used as a control. siRNA delivery was carried out in serum-free media according to the manufacturer’s instructions. After 72 h of incubation with siRNA, the cells were processed for various downstream assays, including RNA and protein analyses.

### Lentivirus preparation

The lentiviral particles were generated using a second-generation packaging system. HEK293T cells were simultaneously transfected with three plasmids: a transfer plasmid (pLKO.1-shRNA for knockdown), packaging plasmid psPAX2 (Addgene, #12260), and envelope plasmid pMD2. G (Addgene, #12259). Co-transfection was performed using Lipofectamine 2000 (Invitrogen, #11668019), according to the manufacturer’s guidelines. Approximately 12 h after transfection, the cell culture medium was replaced with fresh DMEM supplemented with 10% fetal bovine serum and without antibiotics. The viral supernatant containing the newly produced lentiviral particles was collected 48 h later. To remove cellular debris, the supernatant was then centrifuged and filtered through a 0.2 μm polyethersulfone filter, as described in our previous research (Bisserier and Wajapeyee 2018). The effectiveness of gene knockdown or overexpression was confirmed by immunoblotting and quantitative reverse transcription PCR (RT-qPCR), respectively, to evaluate changes in protein and mRNA expression levels.

### In vitrocell proliferation

Cells were fixed with 4% paraformaldehyde for 15 min and rinsed with PBS. Cell proliferation was assessed by immunofluorescence staining for two established markers, Ki67 (Thermo Fisher, Cat# MA5-14520) and proliferating cell nuclear antigen (PCNA) (Thermo Fisher, Cat# PA1-38424). Cell nuclei were counterstained with DAPI (Thermo Fisher, Cat# 62248) to facilitate total cell counts. Following primary antibody incubation, cells were incubated for 1 h at room temperature, protected from light, with species-specific secondary antibodies diluted 1:500 in blocking solution. For Ki67 detection, we used Alexa Fluor Plus 594-conjugated goat anti-rabbit IgG (H + L), highly cross-adsorbed (Invitrogen, Cat# A32740); for PCNA, Alexa Fluor Plus 488-conjugated goat anti-rabbit IgG (H + L), highly cross-adsorbed (Invitrogen, Cat# A32731) was used. Coverslips were mounted using VECTASHIELD Antifade Mounting Medium (Vector Laboratories). Proliferation was quantified as the percentage of Ki67- or PCNA-positive cells relative to total DAPI-stained nuclei per field, using the Tonga bioimage analysis toolbox. Nuclear segmentation and positivity thresholds were defined using the built-in protocols for fluorescence intensity measurement, with a minimum of five fields per condition analyzed in a blinded fashion. All conditions were tested in triplicate, with a minimum of 2,500 cells analyzed per condition.

### MTT assay

MTT assay (Sigma-Aldrich, Cat# M2128) was used to evaluate cell viability and proliferation. After 72 h of treatment, 10 µL of MTT reagent (5 mg/mL) was added to each well containing 100 µL of culture medium. The cells were incubated at 37 °C for 4 h. Next, 100 µL of the solubilization solution (e.g., DMSO) was added to dissolve the formazan crystals. The absorbance was measured at 570 nm using a microplate reader.

### Immunofluorescence and image quantification

Cells were fixed in 4% paraformaldehyde for 15 min, permeabilized with 0.1% Triton X-100 for 10 min, and blocked in a solution of 0.1% Triton X-100 and 5% BSA for 1 h at room temperature. Primary antibodies against H3K27ac and total Histone H3 (see Supplemental Table 1) were applied overnight at 4 °C. After washing, cells were incubated for 1 h at room temperature with species-specific secondary antibodies diluted 1:500 in blocking buffer: Alexa Fluor Plus 594-conjugated goat anti-rabbit IgG (H + L), highly cross-adsorbed (Invitrogen, Cat# A32740) for H3K27ac, and Alexa Fluor Plus 488-conjugated goat anti-mouse IgG (H + L), highly cross-adsorbed (Invitrogen, Cat# A32731) for total H3. Nuclei were counterstained with DAPI (Thermo Fisher, Cat# 62248), and coverslips were mounted using VECTASHIELD Antifade Mounting Medium (Vector Laboratories). For all paired vehicle versus A-485 conditions, immunofluorescence imaging was performed in a single session using identical acquisition parameters, including objective lens, illumination intensity (laser power), detector gain and offset, exposure time, and pixel binning. Pixel saturation was systematically avoided, and linear display ranges were applied uniformly across all images. Quantification was conducted in ImageJ by an investigator blinded to experimental conditions. Nuclei were segmented based on DAPI staining to define per-cell regions of interest. Following uniform background subtraction, mean nuclear intensities for H3K27ac and total H3 were extracted. A per-cell H3K27ac/total H3 intensity ratio was then calculated to normalize for histone abundance. For each condition, a minimum of five non-overlapping fields were analyzed per biological replicate, with all experiments performed at least in triplicate (independent biological replicates). RGB intensity profiles were generated in ImageJ by drawing a defined line across individual nuclei and extracting fluorescence signal intensities for H3K27ac (red), total H3 (green), and DAPI (blue) channels along the line.

### Western blot

Proteins were extracted from the cells and tissues using RIPA buffer (Invitrogen, Cat# 89901) supplemented with protease and phosphatase inhibitors (Thermo Fisher, Cat# A32959). Lysates were clarified by centrifugation at 15,000 × g for 20 min at 4 °C. Protein concentrations were determined using the BCA assay (Thermo Fisher, Cat# A55865). Equal amounts of protein (30 µg) were resolved by SDS-PAGE and transferred to nitrocellulose membranes using a Trans-Blot Turbo system (Bio-Rad). The membranes were blocked with 5% non-fat milk or BSA in TBS-T (0.1%) for 1 h at room temperature and incubated overnight at 4 °C with primary antibodies (listed in Supplemental Table 1). HRP-conjugated secondary antibodies (Cell Signaling Technology) were applied for 1 h at room temperature. Signal detection was performed using enhanced chemiluminescence (Thermo Fisher, Cat# 34075), and the bands were visualized using an iBright imaging system (Invitrogen). Densitometric quantification was performed using ImageJ software.

### Quantitative real-time PCR

Total RNA was isolated from whole lung tissues and PAECs using TRIzol reagent (Invitrogen, Cat# 15596018), according to the manufacturer’s instructions. RNA concentration and purity were verified using a spectrophotometer. One microgram of RNA was reverse-transcribed using the qScript cDNA Synthesis Kit (Quantabio, Cat# 95217). qRT-PCR was performed in technical triplicate on a QuantStudio 6 Pro system (Applied Biosystems) with SYBR Green Master Mix (Thermo Fisher, Cat# A46112). Gene-specific primers (Supplemental Table 1) were used, and amplicon specificity was confirmed using melting curve analysis. Transcript levels were quantified using the ΔΔCt method with normalization to 18S rRNA. Balloon plots and heatmaps were generated using SRPLOT online tool (http://www.bioinformatics.com.cn/srplot) (Tang et al. 2023).

### Public dataset reanalysis

Transcriptomic data were obtained from a previously published cohort of human lung samples (GSE113439), comprising 15 patients with pulmonary arterial hypertension (PAH) and 11 non-diseased controls (Mura et al. 2019). Briefly, fresh-frozen lung tissues were collected at the time of lung transplantation for PAH or from tumor-adjacent non-involved parenchyma in patients undergoing resection for localized lung cancer (controls). The PAH cohort included individuals with idiopathic PAH (IPAH; *n* = 6), PAH associated with connective tissue disease (CTD-PAH; *n* = 4), congenital heart disease (CHD-PAH; *n* = 4), and chronic thromboembolic pulmonary hypertension (CTEPH; *n* = 1). Total RNA was extracted and hybridized to Affymetrix microarrays. For the present analysis, we restricted comparisons to IPAH (*n* = 6) versus controls (*n* = 11) to minimize clinical heterogeneity and focus on a well-characterized PAH subtype. Raw counts were normalized and analyzed using the limma package with empirical Bayes moderation. Multiple testing correction was performed using the Benjamini–Hochberg method to control the false discovery rate (FDR). Differential expression was visualized via volcano plots showing –log₁₀(adjusted p) versus log₂ fold change. To identify regulatory chromatin features associated with transcriptional activation, we ranked differentially expressed genes by adjusted p-value followed by absolute log₂ fold change, and selected the top 250 up-regulated genes (FDR < 0.05) for histone mark enrichment analysis. NIH Roadmap Epigenomics histone modification data were queried using Enrichr, a comprehensive gene set enrichment analysis platform (Kuleshov et al. 2016), available at https://maayanlab.cloud/Enrichr. The full ranked gene list is provided in Supplementary Table S2.

### mRNA sequencing and transcriptome data analysis

Total RNA was extracted from PAECs using the RNeasy Mini Kit (Qiagen, Cat# 74106), and the quality was assessed using a NanoDrop spectrophotometer and an Agilent 2100 Bioanalyzer. Only samples with RIN > 7.0, OD260/280, and OD260/230 ratios ≥ 2.0 were used. RNA integrity was confirmed using agarose gel electrophoresis. Libraries were prepared (250–300 bp insert size) and sequenced on the Illumina NovaSeq 6000 platform (150 bp paired-end reads; Novogene, CA, USA). Differential expression analysis was performed using DESeq2 with Benjamini–Hochberg correction for multiple testing. For visualization in the volcano plot, genes were considered differentially expressed if they met both statistical and biological significance thresholds and an absolute log₂ fold change ≥ 1.5. For heatmap generation, counts were logCPM-normalized, and Z scores were standardized across the top 2,500 most variable genes. Hierarchical clustering and heat maps were generated using Clustergrammer (http://amp.pharm.mssm.edu/clustergrammer/) (Torre et al. 2018). Gene set enrichment analysis (GSEA) was performed using the GSEA software (Broad Institute) to determine whether predefined gene sets exhibited statistically significant, coordinated expression differences between A-485–treated and vehicle-treated PAH-PAECs; enrichment scores and significance were calculated using the default weighted scoring scheme with 1,000 gene set permutations.

### Statistical analysis

Statistical analyses were performed using the GraphPad Prism software (v10.3.0). Data are presented as mean ± SEMs from at least three independent biological replicates. Normality was assessed using the Shapiro–Wilk test. For comparisons between two groups, unpaired two-tailed t-tests or Mann–Whitney U tests were used, as appropriate. One-way ANOVA with Tukey’s or Dunnett’s post-hoc test was used for multi-group comparisons. For non-parametric datasets, the Kruskal–Wallis test with Dunn’s test was used. Statistical significance was set at p < 0.05.

## Results

### EP300 and histone acetylation marks are enriched in lungs from PAH patients and experimental models

**Fig. 1:**
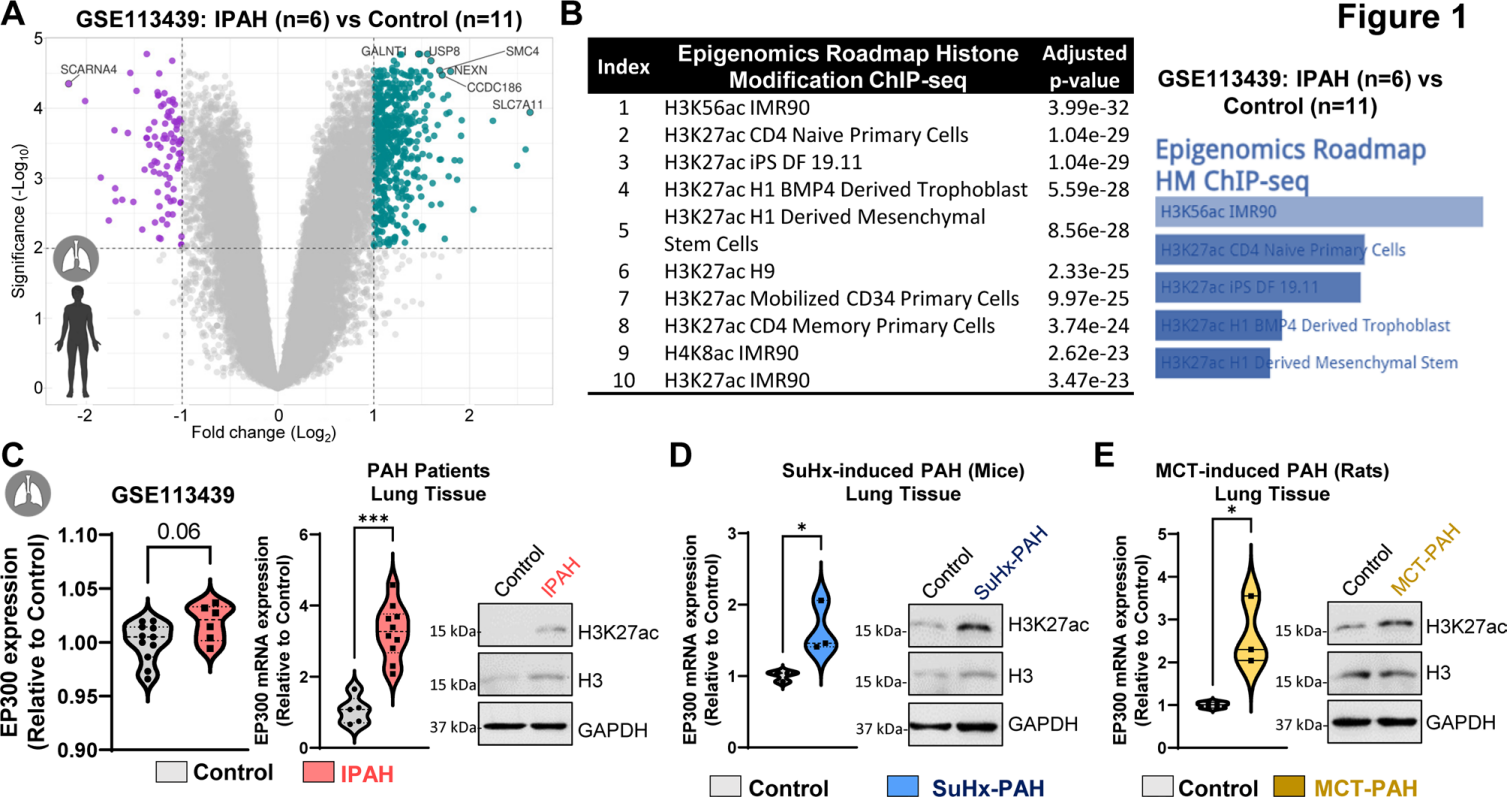
EP300 and H3K27ac enrichment are conserved features of human and experimental PAH. **A** Volcano plot illustrating differentially expressed genes were identified from bulk RNA-seq of human PAH lung tissues (GSE113439; *n* = 6 PAH, *n* = 11 controls), and **B** the top 250 DEGs were analyzed for chromatin mark enrichment using the NIH Roadmap Epigenomics dataset. **C** *EP300* expression was quantified in the dataset GSE113439 (left panel) and validated by qPCR (middle panel) in lung tissues from PAH patients and failed donors obtained from PHBI, used as control (*n* = 5–10 per group). Western blot analysis (right panel) of EP300 and H3K27ac in patient lung lysates. **D-E** EP300 and H3K27ac levels were measured by qPCR and Western blot in lungs from Sugen/hypoxia (SuHx) mice (*n* = 3) and monocrotaline (MCT)-treated rats (*n* = 3). In the SuHx mouse model, normoxic mice served as controls, and vehicle-treated rats were used as controls for the MCT rat model. GAPDH and histone H3 were used as loading controls. Data are presented as the means ± SEMs. *p < 0.05; ***p < 0.001

Given prior evidence of elevated EP300 expression and H3K27ac enrichment in the lungs of PAH patients, we first sought to independently validate these findings and contextualize them within a broader chromatin regulatory framework. Reanalysis of publicly available transcriptomic data (GSE113439; IPAH, *n* = 6 vs. controls, *n* = 11) confirmed widespread transcriptional dysregulation in PAH lungs (Fig. 1A). Integrating these data with NIH Roadmap Epigenomics annotations revealed that top differentially expressed genes are preferentially enriched for active enhancer marks (Fig. 1B). Our analysis revealed that the top 10 histone modifications linked to DEGs in PAH patients were exclusively acetylation marks, including H3K27ac, H3K56ac, and H4K8ac, which are histone lysine residues targeted by the histone acetyltransferase EP300 (Fig. 1B). We next queried the same RNA-sequencing dataset and observed strong upregulation of EP300 in the lungs of patients with IPAH compared to controls (Fig. 1C, left panel). We experimentally validated this observation by qPCR and confirmed a significant increase in EP300 in lung tissue samples from PAH patients compared to controls (Fig. 1C, right panel). This upregulation was accompanied by an increase in global H3K27 acetylation as assessed by immunoblotting (Fig. 1C, right panel). These findings are consistent with prior studies reporting elevated EP300 and H3K27ac enrichment in the lungs of patients with PAH. To determine whether these epigenetic alterations were recapitulated in experimental PAH, we analyzed the lung tissues from Sugen/hypoxia-exposed mice and monocrotaline (MCT)-treated rats (Fig. 1E, F). Both models exhibited increased EP300 mRNA and protein expression, accompanied by elevated H3K27ac levels (Fig. 1E, F). Consistent with lung data, PAH-PAECs displayed higher EP300 and global H3K27ac than FD-PAECs (Supplementary Figure S1). Together, these results established a conserved enrichment of EP300 and its associated histone acetylation marks in both human and experimental PAH, suggesting a central role for EP300-mediated chromatin remodeling in PAH onset and/or progression.

### Pharmacologic inhibition of EP300 attenuates H3K27ac, endothelial proliferation, and oxidative stress in PAH-derived endothelial cells

**Fig. 2:**
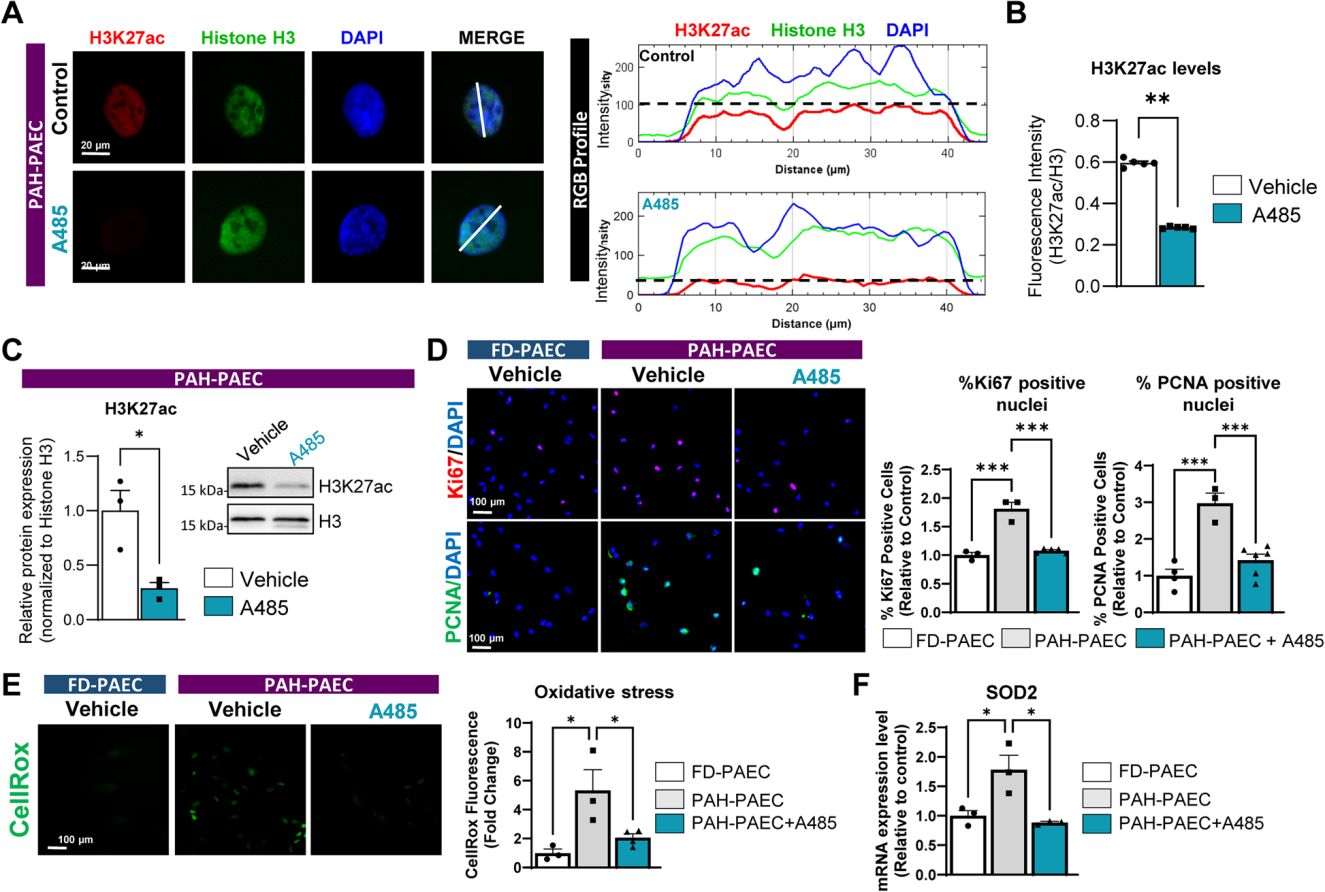
EP300 inhibition suppresses H3K27ac, proliferation, and oxidative stress in PAH-derived endothelial cells. **A** PAH-PAECs were treated with vehicle (DMSO) or A-485 (10 μM, 72 h), and global H3K27ac levels were visualized by immunofluorescence staining for H3K27ac (red), total histone H3 (green), and DAPI (blue), left panel. Representative merged images are shown. Right panel displays RGB intensity profiles across defined nuclear regions in the indicated conditions, illustrating the relative distribution of H3K27ac and total H3 signals. Scale bar = 20 μm. **B** Quantification of the H3K27ac/H3 fluorescence intensity ratio, *n* = 5. **C** Western blot analysis of H3K27ac following A-485 treatment in PAH-PAECs (*n* = 3). **D** Failed donor (FD)-PAECs and PAH-PAECs were immunostained for Ki67 (red) and PCNA (green) following A-485 treatment (10 μM, 72 h); quantification is shown in the right panel (*n* = 3–6). Nuclei were counterstained with DAPI (blue). Scale bar = 100 μm. **E** Oxidative stress levels were measured using CellROX reagent in FD-PAECs and PAH-PAECs ± A-485 (10 μM, 72 h); representative images and quantification data are shown in the right panel (*n* = 3–4). Scale bar = 100 μm. **F** SOD2 transcript levels were assessed by qPCR under the same conditions (*n* = 3). Data are presented as the means ± SEMs. *p < 0.05; **p < 0.01; ***p < 0.001

To further elucidate the mechanistic role of EP300 upregulation and its association with H3K27 acetylation in PAH-PAECs, we next examined its functional contribution to endothelial dysfunction. Evaluation of the efficacy of EP300 inhibition of histone acetylation by immunofluorescence staining showed that A-485 treatment markedly reduced the nuclear H3K27ac signal intensity in PAH-PAECs (Fig. 2A-B). These findings were confirmed by immunoblotting, which confirmed a significant decrease in global H3K27 acetylation following a 72-h treatment with A-485 (Fig. 2C). As A-485 engages the CBP/EP300 HAT domain; hence, the observed drops in H3K27ac further confirmed CBP/EP300 HAT blockade.

Because EP300 is a known regulator of cell cycle progression, we assessed its role in endothelial cell proliferation. Immunostaining for proliferation markers Ki67 and PCNA revealed a significant reduction in the number of proliferating PAECs upon A-485 treatment, indicating that EP300 inhibition suppressed PAH-PAEC proliferation (Fig. 2D). To evaluate the role of EP300 in oxidative stress, we performed CellROX assays in PAH-PAECs and observed a significant decrease in reactive oxygen species following A-485 treatment (Fig. 2E). This reduction in oxidative stress was consistent with the downregulation of transcript levels of the redox-sensitive transcription factor SOD2, as measured by qPCR (Fig. 2F). Collectively, our results indicate that the pharmacological inhibition of EP300 suppresses H3K27ac, limits endothelial cell hyperproliferation, and reduces oxidative stress in PAH-derived PAECs.

### EP300 inhibition reprograms the inflammatory and angiogenic transcriptome of PAH-derived endothelial cells

**Fig. 3:**
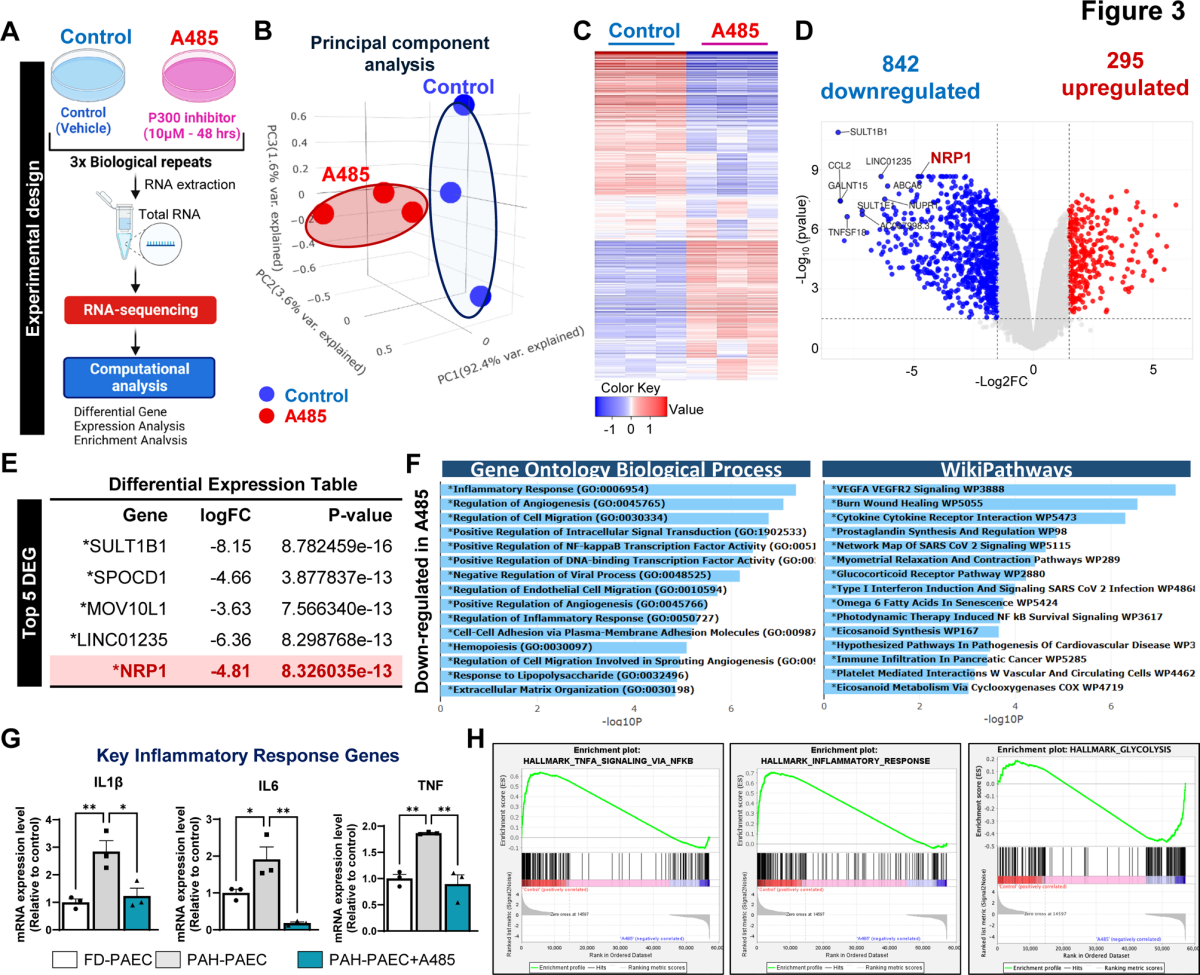
Transcriptomic profiling reveals suppression of angiogenic and inflammatory pathways by EP300 inhibition. **A** RNA-seq was performed on PAH-PAECs treated with vehicle (DMSO) or A-485 (10 μM, 48 h; *n* = 3 biological replicates per group). **B** Principal component analysis was generated to visualize the similarity in gene expression between the two groups: PAECs treated with A-485 (EP300 inhibitor) or DMSO (vehicle, as control). **C** Heatmap of the top 2,500 variable genes from RNA-seq (Z score normalized). **D** Volcano plot depicting 842 downregulated and 295 up-regulated genes (log₂FC ≥ ± 1.5, FDR < 0.01). **E** Identification of NRP1 among the top downregulated genes following A-485 treatment in PAH-PAECs. **F** Gene ontology (GO, left) and WikiPathways (right) analyses of downregulated transcripts. **G** qPCR validation of *IL1B, IL6,* and *TNFA* expression in PAH-PAECs ± A-485 (*n* = 3). **H** GSEA enrichment plots showing the suppression of TNF-α/NF-κB, IL6-JAK-STAT3, and glycolysis-related signatures. Data are presented as the means ± SEMs. *p < 0.05; **p < 0.01

 RNA sequencing was performed in PAH-PAECs following pharmacological inhibition of EP300 with A-485 or vehicle treatment (DMSO), as illustrated in Fig. 3A, to define the transcriptional programs regulated by EP300 in the pulmonary endothelium in the context of PAH. Principal component analysis (PCA) revealed clear segregation between the control and A-485-treated samples, indicating robust transcriptomic reprogramming upon EP300 inhibition (Fig. 3B). Hierarchical clustering and heatmap visualization further demonstrated changes in gene expression, with a predominant pattern of transcriptional repression following A-485 treatment (Fig. 3C). Differential expression analysis revealed 842 downregulated and 295 up-regulated genes in A-485-treated PAH-PAECs (Fig. 3D, Supplementary Table 3). Notably, among the significantly downregulated genes, we identified neuropilin-1 (NRP1), a coreceptor known to modulate VEGFA-VEGFR2 signaling and endothelial permeability, as a transcriptional target of EP300 in PAH-PAECs (Fig. 3E). Our study identified NRP1 as a novel EP300-regulated gene that is enriched for H3K27ac in its regulatory region, suggesting a direct epigenetic mechanism. Its suppression following A-485 treatment suggests a novel axis through which EP300 potentiates endothelial dysfunction in PAH.

To elucidate the biological processes affected by EP300 inhibition, gene ontology (GO) enrichment analysis was conducted on the downregulated transcriptome. Transcriptomic profiling revealed that EP300 inhibition repressed key endothelial pathways implicated in PAH pathogenesis. These include inflammatory signaling, angiogenic regulation, cellular motility, transcriptional activation, and extracellular matrix remodeling, which collectively drive vascular dysfunction and remodeling in the pulmonary vasculature (Fig. 3F, left panel). In line with our previous results, pathway mapping using the WikiPathways database corroborated these findings, revealing a significant downregulation of VEGFA-VEGFR2 signaling, cytokine-cytokine receptor interactions, prostaglandin synthesis, interferon signaling, and glucocorticoid receptor pathways (Fig. 3F, right panel), all of which are known to contribute to vascular inflammation and remodeling in PAH. To validate the anti-inflammatory effect of EP300 inhibition, we performed quantitative PCR analysis, which revealed significant downregulation of canonical pro-inflammatory transcripts, IL1B, IL6, and TNFA, all of which are known to be up-regulated in the lungs of PAH patients (Fig. 3G). Gene set enrichment analysis (GSEA) further confirmed the suppression of hallmark inflammatory pathways, including TNF-α signaling, IL6-JAK-STAT3 signaling, and NF-κB activation, upon EP300 inhibition (Fig. 3H). These results reinforce the concept that EP300 acts as a master chromatin integrator of pro-inflammatory transcriptional programs in the pulmonary endothelium. These findings suggest that EP300 serves as a chromatin-integrated regulator of inflammatory and angiogenic programs in PAH endothelium and identifies NRP1 as a novel EP300-dependent transcript.

### EP300 promotes endothelial dysfunction via H3K27ac-dependent upregulation of NRP1

**Fig. 4:**
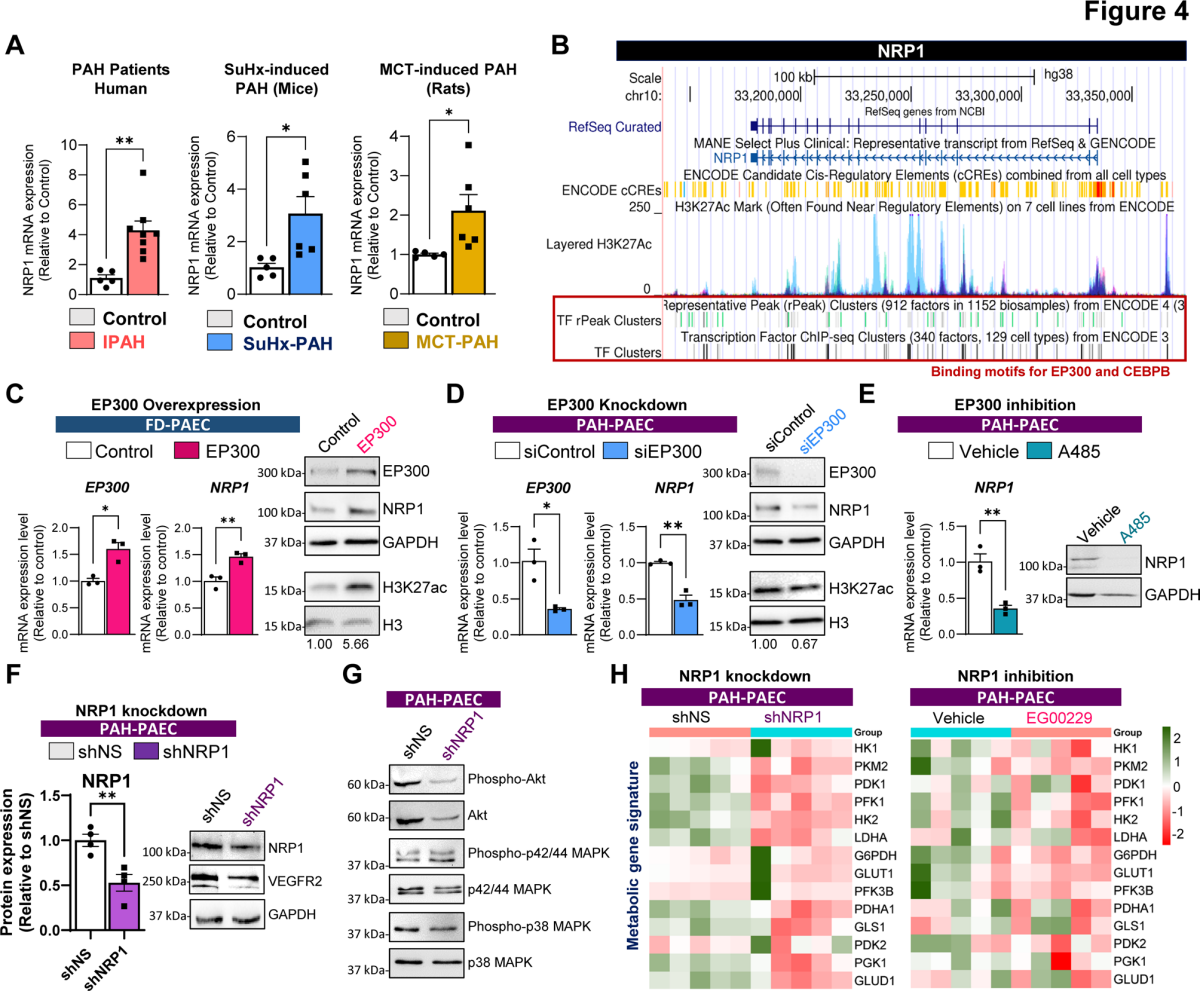
EP300 regulates NRP1 expression via H3K27ac in PAH endothelial cells. **A** qPCR analysis of NRP1 mRNA in lung tissues from PAH patients and non-diseased controls (*n* = 5–8 per group), as well as in two rodent models of PAH (*n* = 5–6). In the Sugen/hypoxia (SuHx) mouse model, normoxic mice served as controls. In the monocrotaline (MCT) rat model, vehicle-treated rats served as controls. **B** ENCODE ChIP-seq tracks showing H3K27ac peaks across the NRP1 locus with predicted EP300 and CEBPB binding sites. **C** EP300 was overexpressed in FD-PAECs via plasmid transfection (72 h); qPCR and Western blotting confirmed increased EP300, H3K27ac, and NRP1 expression (*n* = 3). Densitometry shown below the blots: H3K27ac normalized to H3. **D** EP300 was knocked down in PAH-PAECs using siRNA (72 h); EP300 knockdown was validated by qPCR and immunoblotting and was associated with decreased NRP1 expression (*n* = 3). Densitometry shown below the blots: H3K27ac normalized to H3. **E** A-485 treatment (10 μM, 72 h) reduced *NRP1* transcript and protein levels in PAH-PAECs, as measured by qPCR (*n* = 3) and representative immunoblotting. **F** NRP1 knockdown using siRNA (for 72 h) reduced VEGFR2 expression in PAH-PAECs (*n* = 4). **G** Western blot analysis revealed decreased phosphorylation of AKT, p42/44 MAPK, and p38 MAPK following NRP1 knockdown. **H** PAH-PAECs were treated with the NRP1 inhibitor EG00229 (5 μM, 72 h) or shNRP1 (72 h), and the expression of genes related to mitochondrial metabolism (*HK1, PKM2, PDK1, PFK1, HK2, LDHA, G6PDH, GLUT1, PFK3B, PDHA1, GLS1, PDK2, PGK1,* and *GLUD1*) was assessed by qPCR (*n* = 5). Data are presented as the means ± SEMs. *p < 0.05; **p < 0.01

RNA-seq analysis revealed that EP300 inhibition downregulates NRP1, a key modulator of VEGFA-VEGFR2 signaling. To determine whether NRP1 contributes functionally to endothelial dysfunction in PAH, we assessed its role in promoting proliferative and oxidative phenotypes in PAECs. We also investigated whether EP300 controls NRP1 expression through histone acetylation–dependent transcriptional regulation. We first assessed NRP1 transcript expression in lung tissues from patients with PAH and two established rodent models of experimental PAH: monocrotaline-treated rats and Sugen/hypoxia-exposed rats. In both human and animal models, NRP1 was significantly up-regulated compared to healthy controls (Fig. 4A). Similarly, primary PAH-PAECs exhibited significantly elevated NRP1 mRNA and protein levels relative to FD-PAECs, confirming upregulation of NRP1 in the pulmonary endothelium of PAH patients (Supplementary Figure S2). These findings suggested that elevated NRP1 expression is a conserved feature of PAH pathogenesis.

To explore whether NRP1 is directly regulated at the chromatin level, we used publicly available ENCODE ChIP-seq datasets and identified multiple H3K27ac peaks enriched within the NRP1 promoter and gene body, overlapping predicted binding motifs for EP300 and CEBPB (Fig. 4B). Our computational analysis suggests a potential epigenetic mechanism through which EP300 might activate NRP1 transcription. We next tested our hypothesis by overexpressing EP300 in healthy donor-derived PAECs, which resulted in the induction of EP300 mRNA and protein expression (Fig. 4C, left panel). Western blotting and qPCR analyses revealed that EP300 overexpression increased global H3K27ac levels, as well as NRP1 mRNA and protein expression (Fig. 4C, right panel). Conversely, siRNA-mediated knockdown of *EP300* in PAH-PAECs significantly reduced both H3K27 acetylation and NRP1 expression (Fig. 4D). Similarly, NRP1 downregulation was observed upon pharmacological treatment with the EP300 inhibitor A-485 (Fig. 4E). Next, we silenced *NRP1* in PAH-PAECs using a short hairpin RNA (shRNA) against *NRP1* to determine the functional consequences of NRP1 depletion. Knockdown efficiency was confirmed by immunoblotting (Fig. 4F). NRP1 silencing impaired VEGFR2 expression and attenuated the downstream activation of AKT and MAPK signaling, which are key pathways involved in endothelial proliferation and survival (Fig. 4G). Given that gene set enrichment analysis implicated glycolytic pathways among the transcriptional programs regulated by EP300, we next assessed whether NRP1 is also involved in this metabolic axis. Pharmacological inhibition of NRP1 with EG00229 or siRNA-mediated knockdown of NRP1 in PAH-PAECs significantly downregulated the expression of key metabolic genes, including *PKM2* (pyruvate kinase M2, a rate-limiting glycolytic enzyme), *PFK1* (phosphofructokinase-1, a key regulator of glycolysis), *LDHA* (lactate dehydrogenase A, involved in anaerobic glycolysis), and *GLUT1* (glucose transporter 1, mediating basal glucose uptake) (Fig. 4H). These findings suggest that NRP1 contributes not only to angiogenic signaling but also to metabolic remodeling in the PAH endothelium. Together, these data demonstrate that EP300 directly activates NRP1 expression via H3K27 acetylation in PAECs and that NRP1, in turn, mediates VEGFR2-dependent signaling and mitochondrial gene expression, highlighting a novel epigenetic axis linking chromatin remodeling to endothelial dysfunction in PAH.

### Pharmacological inhibition of NRP1 attenuates EP300-induced endothelial cell proliferation and oxidative stress

**Fig. 5:**
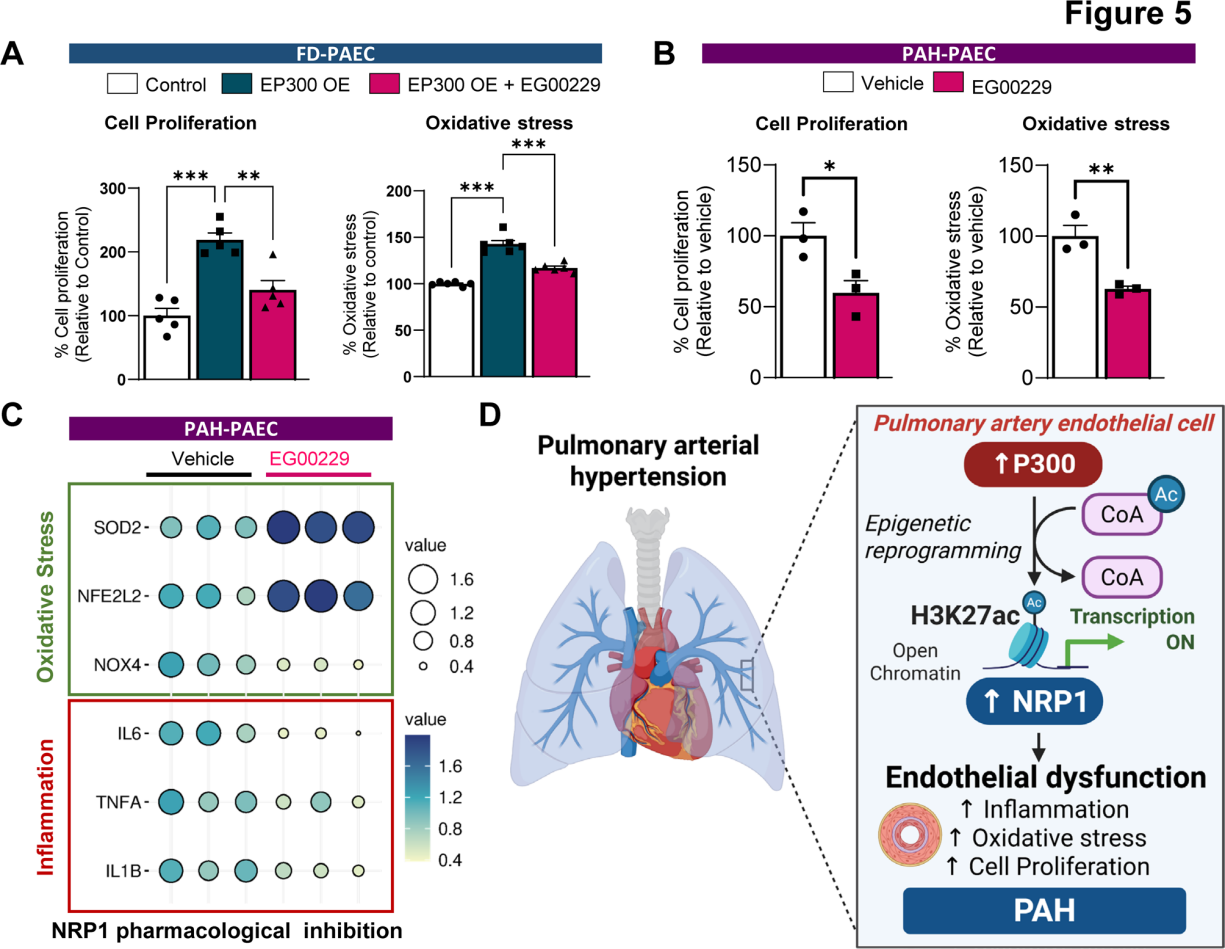
NRP1 inhibition abrogates EP300-driven endothelial proliferation and oxidative stress. **A** FD-PAECs were transfected with a plasmid encoding EP300 or an empty vector alone or were cotreated with ± EG00229 (5 μM, 72 h). Cell proliferation was measured by the MTT assay (*n* = 5), left panel. Oxidative stress levels were assessed by CellROX fluorescence under the same conditions (*n* = 6), right panel. **B** PAH-PAEC were treated either with control vehicle (DMSO) or NRP1 inhibitor EG00229 (5 μM, 72 h). Cell proliferation (left panel) and Oxidative stress levels (right panel) were measured by MTT assay and CellROX fluorescence, respectively (*n* = 3). **C** Oxidative stress markers (*SOD2, NFE2L2, NOX4*) and innflammation markers (*IL6, IL1B, TNFA)* were measured by QPCR, *n* = 3. Data are presented as a balloon plot, generated using the SRPLOT online tool (http://www.bioinformatics.com.cn/srplot). Balloon size reflects absolute fold change, and color indicates directionality and magnitude of regulation. **D** Schematic model illustrating the proposed mechanism whereby EP300 promotes H3K27ac-induced transcriptional activation of NRP1, contributing to endothelial inflammation, oxidative stress, and proliferative dysfunction in PAH. Data are presented as the means ± SEMs. *p < 0.05, **p < 0.01, and ***p < 0.001

 To determine whether NRP1 mediates EP300-induced endothelial dysfunction, we overexpressed EP300 alone or in combination with the NRP1 inhibitor EG00229 in FD-PAECs and assessed endothelial cell proliferation and oxidative stress. EP300 overexpression alone significantly increased endothelial cell proliferation and oxidative stress, as measured by the MTT assay and CellROX fluorescence intensity, respectively (Fig. 5A). Notably, co-treatment with EG00229 markedly attenuated both the pro-proliferative and pro-oxidative effects of EP300, implicating NRP1 as a key downstream effector of EP300-induced endothelial dysfunction. In support, primary PAH-PAECs treated with EG00229 (10 µM, 72 h) exhibited reduced proliferation and decreased intracellular ROS levels (Fig. 5B). Consistent with these phenotypic changes, qPCR analysis revealed that EG00229 treatment downregulated pro-inflammatory and oxidative stress–related genes *(IL6, TNF, IL1B, NOX4*) while up-regulating antioxidant defense genes (*NFE2L2, SOD2*) (Fig. 5C). Together, these findings support a mechanistic model in which EP300 promotes endothelial dysfunction in PAH through epigenetic activation of NRP1.

## Discussion

PAH is increasingly recognized as a multifactorial disorder associated with aberrant epigenetic regulation and sustained transcriptional reprogramming that underlie pathological vascular remodeling. Prior studies have reported elevated EP300 expression and global H3K27 acetylation in PAH lungs; however, the specific role of EP300 in pulmonary endothelial dysfunction has remained largely undefined. Here, we identify EP300 as a key chromatin regulator that promotes endothelial dysfunction in PAH through H3K27ac-dependent activation of NRP1, a coreceptor for VEGFR2 involved in vascular permeability, inflammation, and remodeling. We show that EP300 inhibition, either pharmacologically or genetically, reduces H3K27ac levels, suppresses NRP1 expression, and reverses pathogenic endothelial phenotypes, including oxidative stress, inflammatory signaling, and metabolic reprogramming. These findings provide the first direct evidence that EP300 drives endothelial dysfunction in PAH and position the EP300-H3K27ac-NRP1 axis as a central mechanism in disease initiation and progression.

Our findings are consistent with prior studies demonstrating increased EP300 expression and H3K27ac enrichment in pulmonary fibroblasts and smooth muscle cells from PAH patients (Chelladurai et al. 2022). Notably, Chelladurai et al. demonstrated that EP300 inhibition by RNA interference or small-molecule compounds reduced PAH phenotypes and mesenchymal signatures in arterial fibroblasts and smooth muscle cells (Chelladurai et al. 2022). They further showed that EP300/CBP inhibition attenuates vascular remodeling in rodent models and human PAH lung slices ex vivo (Chelladurai et al. 2022). Consistent with these findings, genome-wide H3K27ac profiling revealed EP300-dependent enhancer activation in VEGF-stimulated endothelial cells (Zhang et al. 2013), whereas Su et al. reported that EP300 inhibition improved endothelial function and vascular stiffness in models of metabolic stress (Su et al. 2020). Our study extends these findings by demonstrating that EP300 similarly modulates H3K27ac deposition in PAECs, driving the transcriptional activation of NRP1 and pro-inflammatory angiogenic programs. Because A-485 targets the shared histone acetyltransferase domain of CBP and EP300, the observed effects reflect inhibition of CBP/EP300 HAT activity. This is consistent with the reductions in H3K27ac in our in vitro models. This study further supports the importance of EP300-H3K27ac dynamics in maintaining pathogenic endothelial phenotypes in PAH patients.

Our study identified NRP1 as a direct and novel target of EP300 in the pulmonary endothelial cells. EP300 promotes H3K27ac deposition at NRP1 regulatory loci, driving H3K27ac-dependent transcription. NRP1 amplifies VEGFR2 signaling, promotes glycolytic gene expression, and contributes to endothelial proliferation and oxidative stress. Consistent with our findings, NRP1 has previously been shown to increase VEGF-induced endothelial permeability by forming complexes with VEGFR2 and facilitating downstream MAPK signaling (Plein et al. 2014). However, its role in PAH remains unclear. Here, we found that NRP1 was up-regulated in multiple preclinical models and human lung samples. Consistent with our findings, NRP1 has also emerged as a circulating biomarker of systemic sclerosis-associated PH (Bauer et al. 2021; Bahi and Li 2024). Notably, previous studies have identified NRP1 as part of an eight-protein serum panel that effectively distinguished PAH patients from non-PH patients using machine learning-based proteomic profiling (Bauer et al. 2021). These findings support its relevance as both an early detection biomarker and potential pathogenic mediator in the initial stages of PAH associated with systemic sclerosis. Our computational analysis revealed H3K27ac enrichment at the NRP1 regulatory loci. Gain- and loss-of-function experiments have confirmed that EP300 transcriptionally activates NRP1, promoting endothelial dysfunction in PAH. Although our primary focus was on endothelial dysfunction, the EP300-NRP1 axis also appears to be involved in PASMCs. Indeed, our results revealed that PAH-PASMCs exhibited elevated EP300 and NRP1 expression, along with increased global H3K27ac levels, compared with FD-PASMCs (Supplementary Figure S3). Interestingly, pharmacological inhibition of EP300 with A-485 significantly reduced NRP1 expression at both the mRNA and protein levels in these cells (Supplementary Figure S4), indicating that EP300 epigenetically regulates NRP1 in PASMCs as well. Overall, these findings suggest that the EP300-NRP1 axis may not be restricted to endothelial cells, but rather extends to other vascular cell types implicated in PAH pathogenesis. Pal et al. demonstrated that NRP1 modulated vascular permeability by regulating VE-cadherin phosphorylation in an organ-specific manner (Pal et al. 2024). Valdembri et al. reported that NRP1 promotes endothelial adhesion to fibronectin via interactions with GIPC1 and α5β1 integrin (Valdembri et al. 2009). More recently, Lemay et al. (Lemay et al. 2025) identified α5β1 integrin as a pivotal driver of vascular remodeling in PAH, demonstrating that its inhibition reverses pathological vascular changes and improves hemodynamics across multiple preclinical models. These findings further support the hypothesis that NRP1 contributes to aberrant extracellular matrix organization in PAH by facilitating integrin-mediated endothelial adhesion and signaling.

In agreement with previous studies, our transcriptomic analyses further revealed that EP300 inhibition downregulated multiple gene networks implicated in angiogenesis, inflammatory signaling, and extracellular matrix remodeling. These include key mediators of the TNF-α, interferon, and cytokine receptor pathways, as well as metabolic regulators, such as GLUT1, PKM2, and LDHA. Collectively, our results suggest that EP300 blockade may restore endothelial function by mitigating pro-inflammatory and metabolic gene programs. Given the dynamic and reversible nature of histone acetylation, our findings underscore the therapeutic potential of targeting EP300 in PAH. In line with our findings, EP300 inhibition has demonstrated efficacy in reversing hemodynamic abnormalities and pulmonary vascular remodeling in experimental PAH (Chelladurai et al. 2022). Our study extends these findings by showing that EP300 inhibition in PAH-derived PAECs attenuates hallmark pathogenic features, including proliferative signaling, oxidative stress, and pro-inflammatory gene expression. Future studies are warranted to determine whether these interventions can restore vascular homeostasis in vivo and confer clinical benefits to PAH patients.

This study has several limitations. First, although EP300 and NRP1 were consistently up-regulated in PAH lungs, PAH-PAECs, and two rodent models (SuHx and MCT), our mechanistic analyses were restricted to in vitro systems. While prior studies have demonstrated that EP300 inhibition attenuates pulmonary hypertension in vivo, these efforts primarily focused on fibroblasts and smooth muscle cells. Our study extends this knowledge by identifying a novel EP300–NRP1 axis in endothelial cells; however, in vivo validation of this signaling pathway in the endothelium remains a critical next step. Future studies directly addressing this limitation through pharmacological and genetic targeting of NRP1 in the pulmonary endothelium of animal models of PAH with comprehensive phenotyping, including echocardiography, hemodynamics, and histopathology, are warranted. Second, while our reanalysis of the GSE113439 dataset validated broad transcriptional dysregulation in IPAH lungs, EP300 itself did not reach FDR significance. This likely reflects the modest sample size, cell-type heterogeneity in whole lung tissue, and elevated EP300 expression in cancer-adjacent control samples. This dataset was primarily leveraged to support integrative analyses with NIH Roadmap Epigenomics annotations, to demonstrate that chromatin-level regulatory insights may be underappreciated by transcriptomic data alone. Third, although our chromatin mark-enrichment screen also identified H3K56ac, we prioritized H3K27ac because validated ENCODE tracks and multiple Roadmap datasets supported robust integrative analyses, whereas H3K56ac, despite a very strong signal, was only identified in a single dataset in our pipeline; nonetheless, H3K56ac remains a promising avenue that we are pursuing in ongoing studies. Fourth, while we demonstrate that EP300 regulates NRP1 via H3K27ac, genome-wide chromatin immunoprecipitation studies are needed to map direct EP300 binding sites and to elucidate the broader enhancer landscape underlying endothelial dysfunction in PAH. Finally, the potential cooperation between EP300-mediated H3K27ac and other chromatin remodelers, bromodomain-containing histone readers, and lineage-defining transcription factors remains unexplored. These interactions represent promising avenues for future mechanistic studies aimed at understanding how EP300-driven epigenetic reprogramming contributes to pulmonary vascular remodeling.

## Conclusion

This study revealed a novel epigenetic mechanism in which EP300 drives pulmonary endothelial dysfunction in PAH through H3K27 acetylation and transcriptional activation of NRP1. Our study suggests that the EP300-NRP1 signaling axis is responsible for metabolic reprogramming in PAH-PAECs. Inhibition of EP300 or NRP1 attenuates the key features of PAH. These findings establish EP300 as a mechanistic driver of endothelial dysfunction in PAH, and highlight the EP300-H3K27ac-NRP1 axis as a promising therapeutic approach to prevent PAH onset or attenuate PAH severity. Given that endothelial dysfunction is a key early driver of pulmonary vascular remodeling, our findings identify EP300-mediated histone acetylation as a critical upstream event that may initiate and exacerbate disease progression. Targeting this epigenetic axis may offer a novel therapeutic strategy to restore endothelial homeostasis and modify disease trajectory in PAH.

## Supplementary Information

Supplementary Material 1.

Supplementary Material 2.

Supplementary Material 3.

## Data Availability

All datasets and materials supporting the findings of this study are available from the corresponding author upon reasonable request.
