# Supplementary material for: Epigenetic reprogramming drives endothelial dysfunction via neuropilin-1 in pulmonary hypertension
Source: Mol Med. 2025 Dec 16;31:336. doi: 10.1186/s10020-025-01386-0 (PMC12709837; doi:10.1186/s10020-025-01386-0)
Supplement: Supplementary file 1 — Supplementary Material 1. [file 10020_2025_1386_MOESM1_ESM.docx]

**Supplementary Table 1.** Primer sequences for RT-qPCR analysis; clone ID and catalog numbers for shRNAs (Open Biosystems) and plasmids; antibodies used; source, and concentration of chemical inhibitors used.

| **Application** | **Gene symbol** | **Species** | **Forward primer (5′-3′)** | **Reverse primer (5′-3′)** |
| --- | --- | --- | --- | --- |
| **RT-qPCR** | EP300 | Human  Mouse  Rat | CTAATTCTCTCTCCAATCAAGTGCG | TGGCAAAATGCCCTTGTTCCAT |
|  | G6PDH | Human | GGTGACCTGGCCAAGAAGAA | GGG CAT TCA TGT GGC TGT TG |
|  | GLS1 | Human | AGAAGGCACAGACATGGTTGG | CCCAGCAACTCCAGATTTTGC |
|  | GLUD1 | Human | GGAGATGTCC TGGATCGCTG | CTCATAGAGTGCAGGCCCAC |
|  | GLUT1 | Human | GCAGATGATGCGGGAGAA GAA | GTGAAGGCCGTGTTGACGATA |
|  | HK1 | Human | ATGAAGAATGGCCTCTCCCG | CACGATGTTCTCTGGGGTGT |
|  | HK2 | Human | AGAGCATCCTCCTCAAGTGGA | TGAGGCAACTTCACAGTGAG |
|  | IL1β | Human | ATGATGGCTTATTACAGTGGCAATG | ATCTTCCTCAGCTTGTCCATGG |
|  | IL6 | Human | ACAAGAGTAACATGTGTGAAAGCAG | ACTCTCAAATCTGTTCTGGAGGTAC |
|  | LDHA | Human | CAG TTG TTG GGG TTG GTG CT | TGT TCA CGT TTC GCT GGA CC |
|  | NFE2L2 | Human | TGCCCCTGGAAGTGTCAAACA | GGCTTGAATGTTTGTCTTTTGTGAATGG |
|  | NRP1 | Human  Mouse  Rat | TTTGGCTGGGGCTCTCACA | TAGATGAAGTTGCCATCTCCTGT |
|  | NOX4 | Human | AGAGCTACGCACAGCAGCT | CAAAGCTCTCTCCATTCTCCGG |
|  | PDHA1 | Human | CGTG GTTT TCTGTC ACTTGTGTG | GAAGTTCTTGGCGTACATGTGC |
|  | PDK1 | Human | ATGAAGCAGTTCCTGGACTTCG | CGGATGGGGTCC TGAGAAGATT |
|  | PDK2 | Human | TGCAGAGCTGGTATGTCCAGA | TCTGGTTGGAGACTGGGTCAT |
|  | PFK1 | Human | GATGGGTGTGGAAGCAGTGA | TTGTTCATGAAGCTCCGGCC |
|  | PFK3B | Human | CCAGCCCGGATTACAAAGACT | GCACGTGGATGTTCATCAGGT |
|  | PGK1 | Human | GAACTCAAATCTCTGCTGGGCA | AGAAGCATCTTTTCCCTTCCCTT |
|  | PKM2 | Human | GAGAACATCCTGTGGCTGGA | CTGCTCCACCCCAA ACTTCA |
|  | SOD2 | Human | AACGCGCAGATCATGCAGCT | TTCAGTGCAGGCTGAAGAGC |
|  | TNFα | Human | CTTGTTCCTCAGCCTCTTCTCCTTC | GGGTTCGAGAAGATGATCTGACTG |
|  | 18S | Human  Mouse  Rat | GTAACCCGTTGAACCCCATT | CCATCCAATCGGTAGTAGCG |
|  | | | | |
| **shRNAs** | **Gene symbol** | **Clone ID** | | **Catalog number** |
|  | *NRP1* | TRCN0000063523 | | RHS3979- 201781220 |
|  |  |  | |  |
| **Overexpression** | **Gene symbol** | **Clone ID** | | **Catalog number** |
|  | *EP300* | / | | MHS1010-202699710 |
|  | | | | |
| **Immunoblotting** | **Protein symbol** | **Antibody source** | | **Dilution** |
|  | EP300 | Cell signaling | | 1:1000 |
|  | H3K27ac | Cell signaling | | 1:1000 |
|  | Histone H3 | Cell signaling | | 1:1000 |
|  | NRP1 | Cell signaling | | 1:1000 |
|  | p-VEGFR2 | Cell signaling | | 1:1000 |
|  | Tot-VEGFR2 | Cell signaling | | 1:1000 |
|  | GAPDH | Invitrogen | | 1:5000 |
|  | | | | |
| **Pharmacological agents** | **Compounds** | **Concentration** | | **Source** |
|  | A485 | 2 µM | | MedChem Express |
|  | EG00229 | 5 µM | | MedChemExpress |
